# Spider silk felting—functional morphology of the ovipositor tip of *Clistopyga* sp. (Ichneumonidae) reveals a novel use of the hymenopteran ovipositor

**DOI:** 10.1098/rsbl.2016.0350

**Published:** 2016-08

**Authors:** Niclas R. Fritzén, Ilari E. Sääksjärvi

**Affiliations:** Department of Biology, Zoological Museum, University of Turku, 20014 Turku, Finland

**Keywords:** Hymenoptera, spider parasitoid, functional behaviour, ovipositor use

## Abstract

Apical serrations of the hymenopteran ovipositor have been widely postulated to originally constitute adaptations for cutting through hard substrates. Simplifications of the ovipositor tip have occurred in several ichneumonid wasp genera associated with spiders. Despite such reduction in *Clistopyga* (Hymenoptera, Ichneumonidae), the ovipositor still possesses some apical serrations. Through the first detailed study, we believe, on the behaviour of an ovipositing *Clistopyga* species, we show that it can alter its ovipositor for different purposes and that the primary function of the apical serrations is clinging to its spider host as the spider attempts to escape. Intriguingly, we also discover a hitherto undocumented adaptation for the hymenopteran ovipositor. The female wasp seals openings in the silken spider nest by using its ovipositor on the silk in a highly sophisticated way that is comparable to how humans entangle wool by needle felting. By studying the ovipositor morphology through a scanning electron microscope, we elucidate how this works, and we hypothesize that by closing the nest the female wasp protects its developing kin.

## 1. Introduction

Female wasps typically have an ovipositor for inserting eggs into plant tissues or insect hosts. In some groups, oviposition occurs direct from the genital opening and the ovipositor functions mainly as a venom stinger. In nearly all Ichneumonoidea, one of the superfamilies of parasitoid wasps including the families Ichneumonidae and Braconidae, the ovipositor serves a similar set of functions. These are navigating or penetrating the substrate, locating and assessing the host, piercing and staying in the host, injecting venom and finally laying an egg [1]. In addition, the ovipositors can be used for infanticide by killing or removing eggs and larvae from already parasitized hosts prior to oviposition [2]. Based on their life strategy, parasitoid wasps can usually be divided into two broad categories: idiobionts and koinobionts [3]. Idiobionts kill or permanently paralyse their hosts at the moment of attack, whereas koinobionts allow their hosts to recover normal activity for a time after the oviposition. *Clistopyga* Gravenhorst, 1829 (Hymenoptera, Ichneumonidae, Pimplinae) is a medium-sized genus currently comprising nearly 50 described species [4]. Most parasitoid wasps attack other insects, but little is known of the life-history strategies of *Clistopyga*. A few *Clistopyga* species have been reared from spider nests containing egg sacs [5,6], and there is a single record of a species reared from a cocoon found beside the dead body of a jumping spider (Salticidae) [7,8], indicating that at least some of the species act as idiobiont or even koinobiont ectoparasitoids of spiders [8,9]. What makes *Clistopyga* especially interesting is that it has been proposed to demonstrate the evolutionary transition from idiobiont ectoparasitoid wasps that parasitized silken cocoons of Lepidoptera to groups laying eggs in silken egg nests of spiders to the more derived groups that develop as koinobiont ectoparasitoids on mobile spiders [7,8,10]. In addition, our recent fieldwork in the tropics has revealed a plethora of different *Clistopyga* ovipositor shapes, suggesting that oviposition and other use of the ovipositor would be of great interest when trying to understand their evolution. Our detailed studies on a northern European *Clistopyga* species have given a completely new insight into the life-history strategies of this highly interesting genus. The studied species is an idiobiont ectoparasitoid of jumping spiders (NR Fritzén, in preparation). It attacks and permanently paralyses the host within its silken nest. In this paper, we concentrate on the functional morphology of the ovipositor tip in the ovipositing female.

## 2. Material and methods

The single *Clistopyga* female used for the study was collected as a minute larva on a paralysed adult female *Salticus cingulatus* (Panzer, 1797) jumping spider in its silken nest under loose bark on a sun-exposed pine tree (*Pinus sylvestris*) on a sea shore in western Finland (63.159° N 21.309° E) on 30 August 2015. In the nest, there were both unhatched eggs and a few hatched spiderlings. The larva feeding on the spider female was reared *in vitro*. The identity of the species is at this time unclear and it is therefore referred to as ‘*Clistopyga* sp.’. All figures and the electronic supplementary video are of this female. It is deposited in the Zoological Museum, University of Turku (ZMUT).

**Figure 1.**
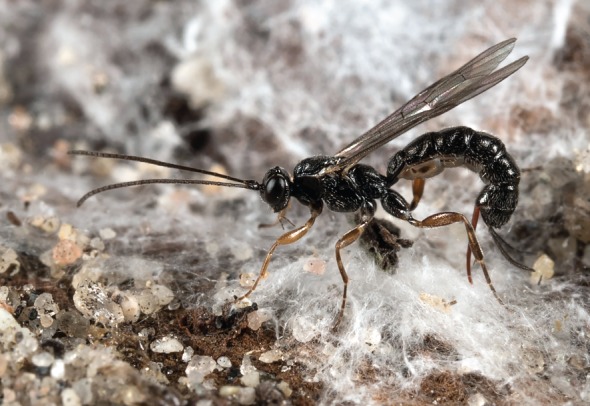
*Clistopyga* sp. in action. With its ovipositor, it searches for the host, stings, clings to and paralyses it, lays an egg and finally seals any opening in the silken spider nest by using it as a minute felting needle. (Online version in colour.)

**Figure 2.**
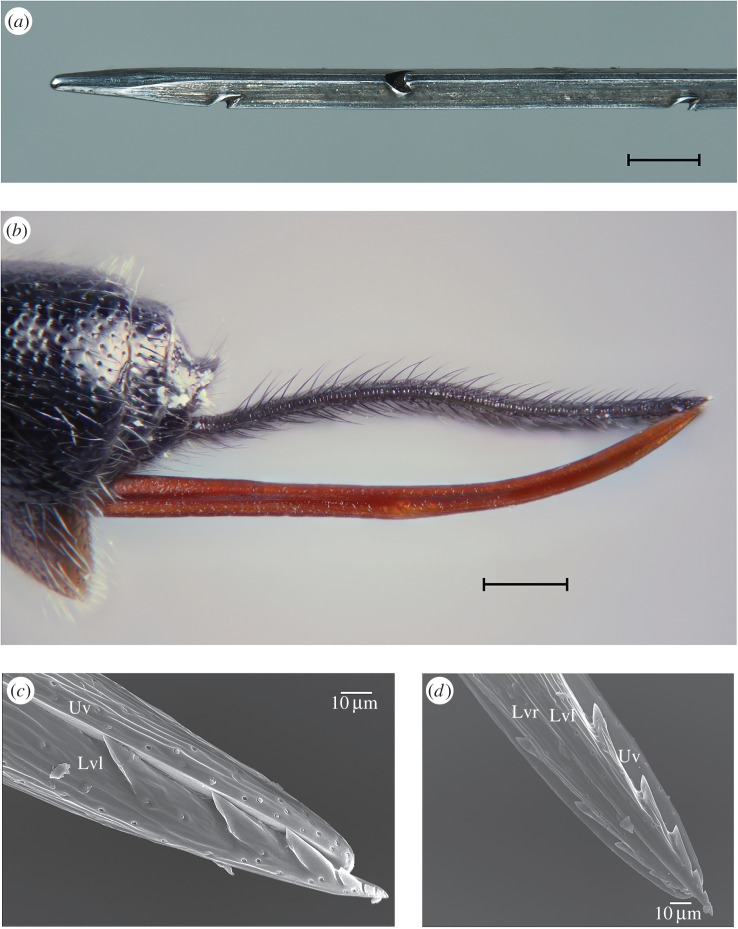
A felting needle and the ovipositor of *Clistopyga* sp. with a similar function. (*a*) The tip of a felting needle with procurved notches that entangle fibres during the downstroke. Scale bar, 1 mm. (*b*) Stacked image of the apically upcurved ovipositor. Scale bar, 0.2 mm. (*c*) SEM image of the ovipositor tip in lateral view showing six recurved apical teeth of the left lower valve, which are diminishing in size towards the apex of the ovipositor. When used as a felting needle, the tips of the movable lower valves need to be held beyond the tip of the upper valve in order to expose the teeth. Because the teeth are recurved, the ovipositor entangles spider silk only during the upstroke. (*d*) The same tip in ventral view showing how the teeth can be enclosed by the broader upper valve when not in use. Uv, upper valve; Lvl, left lower valve; Lvr, right lower valve. (Online version in colour.)

*Salticus cingulatus* mainly lives on tree trunks, where it makes silken retreats under bark, in which it rests, moults, lays eggs and overwinters. To study the behaviour of the wasp, small pieces of bark were detached from trees and collected if containing nests with the host species inside. At the time of collection, late September to October, they constituted mainly subadult individuals that were about to hibernate in the nests, but also a few adult females (without egg sacs). These were offered to the wasp in Petri dishes. In order to prevent the spider from escaping attack, some specimens were chilled for a while in a freezer or the attempt was stopped by tweezers. The behaviour of the wasp encountering the nests was studied through an Olympus SZX16 microscope provided with an Olympus OM-D EM-5 mark II camera that video-documented the behaviour in full HD at a rate of 50 f.p.s.; figure 1 was made with the same camera with TF-22 twin flash. The stacked image of the ovipositor (figure 2*b*) was taken in ZMUT, using a Canon EOS 7D digital camera attached to an Olympus SZX16 stereomicroscope. Scanning electron microscope images (figure 2*c–d*) were taken in ZMUT, using a Jeol JSM-5200.

## 3. Results

### (a) Morphology of the ovipositor tip

The ichneumonoid ovipositor comprises a single upper valve and a pair of lower valves [1]. The outer surfaces of the ovipositor in *Clistopyga* sp. lack serrations and nodi (figure 2*b*). Dorsally at the apex of each lower valve, there are six recurved teeth diminishing in size towards the apex of the ovipositor (figure 2*c*). These teeth are enclosed by the broader upper valve (figure 2*d*) when the tips of the independently movable lower and upper valves are kept aligned, i.e. when at rest.

### (b) Functional morphology

The serrations at the apex of the lower valves show adaptations for three functional behaviours of the ovipositing female. (i) Solely by inserting the ovipositor tip into the host the female wasp clings to the struggling spider and prevents it from escaping during envenomation. (ii) With the aid of the ovipositor tip reinserted into the host after a successful paralysation, the female wasp performs physical manipulation of the host by repositioning the spider into a favourable posture for oviposition. (iii) Finally, after a successful oviposition, *Clistopyga* sp. instantly begins to stab the silk with its ovipositor around the site through which oviposition was accomplished. The stitching is usually done with zigzagging movements with the ovipositor alternately angled against the surface of the silk. Under the studied *in vitro* conditions, the average time used for stitching (*n* = 11) was 3 min 10 s (*s* = 1 min 38 s). By this stitching, the female wasp closes openings (made by the spider or the attacking wasp itself) of the nest (see electronic supplementary material, video S1) and partly packs other parts of the silken nest. On a few occasions, it was seen that silk attaches to the ovipositor tip which entangles the silk exclusively during the upstroke.

## 4. Discussion and conclusion

Closing and repacking the spider's silken nest involves a functional behaviour very similar to human needle felting, whose principle is the use of a needle with notches along the shaft (figure 2*a*), which grab the top layer of fibres and tangle them with the inner layers as the needle enters sheep wool. Because the notches are procurved, they do not stick to the fibres during the upstroke. In the case of *Clistopyga* sp., the ovipositor acts as the felting needle, and openings in the nest are sealed using this method that also makes the fluffy silk stiffer. The zigzagging movements with the ovipositor probably add extra strength to the ‘felt’. The time expended for felting, though performed under rather unnatural conditions, indicates the importance of this behaviour. We can think of four factors selecting for it: (i) protection from subsequent predation or parasitism, (ii) stabilizing microclimatic conditions inside the silken nest, (iii) prevention of any awakening spider from escaping, and (iv) preventing the mobile larval offspring from accidentally leaving the nest. Contrary to human needle felting, the *Clistopyga* sp. ovipositor tangles the silk fibres only during the upstroke. When *Clistopyga* sp. searches for its host inside the spider nest (figure 1), it repeatedly inserts the ovipositor into the silk and withdraws it (NR Fritzén, in preparation). These movements are very smooth, and no silk attaches to the ovipositor tip. This indicates that the female wasp alters the ovipositor for different silk-stabbing purposes. The morphology of the ovipositor shows how this works. Dorsal teeth on the lower valves, instead of serrations at the outer surfaces, of the ovipositor enable the wasp to enclose them with the broader upper valve (figure 2*d*), e.g. during the search for the host. This is possible because the three valves of the ovipositor can slide independently of each other. When the ovipositor is used as a felting needle, and likewise for clinging to the host, the tips of the lower valves are held beyond the tip of the upper valve in order to expose the teeth. The fact that the teeth are recurved explains why the silk is entangled only when the ovipositor exits the silk (i.e. on the upstroke).

Physical manipulation of the host is common in many aculeate hymenopterans but has only rarely been observed among parasitoid wasps [11]. The purpose of the manipulation seen in *Clistopyga* sp. is not to transport the host but only to arrange it better for oviposition. However, there is an observation of a *Clistopyga* on a tree trunk carrying a spider attached to the ovipositor [12].

Apical serrations of hymenopteran ovipositors have been postulated to originally be an adaptation to cutting through hard substrates [1]. If this need is reduced, there have, sometimes, been simplifications in the upper and lower valves [1], typically seen in several ichneumonid genera associated with spiders and their egg sacs [6,13] and well demonstrated by both the dorsal surface of the upper valve and the ventral surface of the lower valves of the ovipositor of *Clistopyga* lacking any apical serrations or protuberances. In all *Clistopyga* species we have examined, dorsal recurved teeth of the lower valves have been seen, though differing greatly in size. Because the plethora of different shapes of *Clistopyga* ovipositors indicates a variety of behavioural adaptations and the common use of the ovipositoral teeth in *Clistopyga* is possibly clinging to a spider (regardless of whether it is used as a feeding source or only attacked in order to get access to its offspring), we doubt that all species will turn out to be felters. However, the original adaptation for these teeth needs further investigation. They often occur together with ventral serrations on the lower valves in combination with a dorsal pre-apical protuberance on the upper valve, at least in other related genera associated with silken spider nests (*Zaglyptus* Foerster and *Tromatobia* Foerster), but also in related groups attacking lepidopteran cocoons (e.g. *Iseropus* Foerster and *Acropimpla* Townes). The use of these teeth for felting spider silk is therefore undoubtedly an extreme exaptation. The presence of dorsal teeth of the ventral valves has attracted little attention in hypotheses of the transitions from ectoparasitism to endoparasitism and vice versa or from idiobiosis to koinobiosis [14]. The presence and especially the use of them in other groups not attacking the spiders themselves should be taken into account in any re-study of the evolution of the groups with a common interest in silk, i.e. the hypothesized transition within Ephialtini (Pimplinae) from lepidopteran idiobiont cocoon parasitoids to those with a koinobiont lifestyle on free-living web-building spiders.

## Data accessibility

A higher resolution version of the supplementary video is available at https://www.youtube.com/watch?v=rwXm_MYZYqo.
